# Beneficial effects of the olive oil phenolic components oleuropein and hydroxytyrosol: focus on protection against cardiovascular and metabolic diseases

**DOI:** 10.1186/s12967-014-0219-9

**Published:** 2014-08-03

**Authors:** Stefania Bulotta, Marilena Celano, Saverio Massimo Lepore, Tiziana Montalcini, Arturo Pujia, Diego Russo

**Affiliations:** 1 Department of Health Sciences, University “Magna Graecia” of Catanzaro, Campus “S. Venuta”, Catanzaro, 88100, Italy; 2 Department of Medical and Surgical Sciences, University “Magna Graecia” of Catanzaro, Campus “S. Venuta”, Catanzaro, 88100, Italy

**Keywords:** Oleuropein, Hydroxytyrosol, Virgin olive oil, Phenols, Antioxidant, Cardiovascular disease, Diabetes mellitus

## Abstract

The overall health beneficial action of olive oil phenolic components is well established. Recent studies have elucidated the biological effects of two isolated compounds, namely oleuropein and hydroxytyrosol, with particular attention on their antioxidant activity. Thus, a protective action has been demonstrated in preclinical studies against several diseases, especially cardiovascular and metabolic disorders.

The present review will describe the biological effects of oleuropein and hydroxytyrosol, with particular attention on the molecular mechanism underlying the protective action on cardiovascular and metabolic alterations, as demonstrated by in vitro and in vivo experimental studies performed with the isolated compounds.

## Introduction

**Figure 1:**
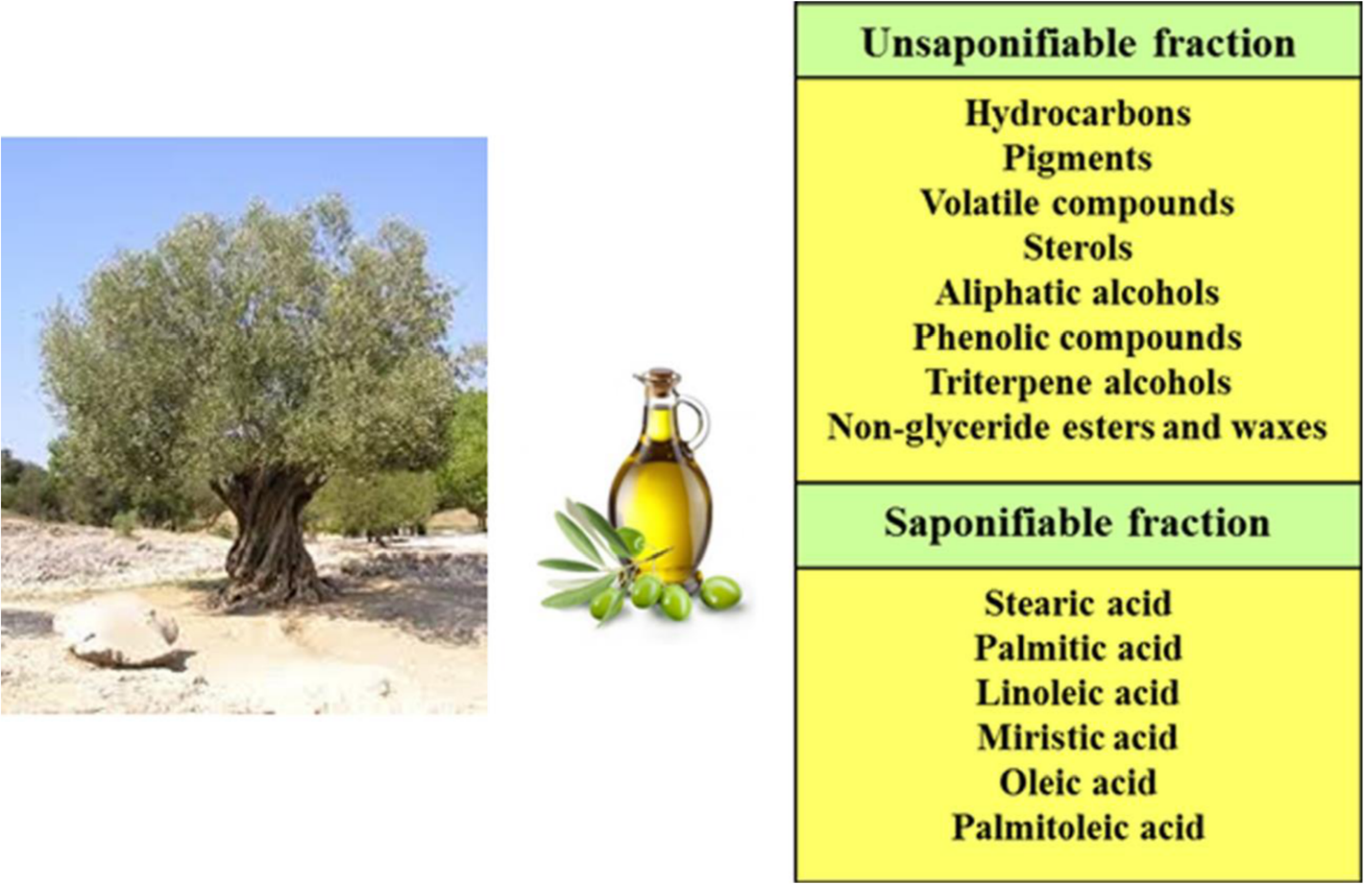
Composition of unsaponifiable and saponifiable fractions of olive oil.

Several studies have assigned to the virgin olive oil (VOO) most of the beneficial effects on human health attributed to the Mediterranean diet [1]-[6]. Initially, the richness of monounsaturated fatty acids (MUFA), and in particular oleic acid, was considered as the major healthful characteristic of VOO. Later on, after the observation that other aliments rich in MUFA, as rapeseeds, soybean and sunflower, were not comparable with VOO as healthful food [7],[8], the role of some ‘minor components’ has been taken into consideration, also because such compounds are able to maintain their biological action when VOO is consumed in crude form. There are more than 200 ‘minor components’ in the unsaponifiable fraction of olive oil, which represent about 2% of the total weight, and include a number of heterogeneous compounds non-chemically related to fatty acids (Figure 1) [9],[10].

Particular attention has been focused on the nutraceutical properties of those compounds provided with antioxidant activity. The most abundant antioxidants in VOO are lipophilic and hydrophilic phenols [11] (Table 1), which are physiologically produced in the plant to react against various pathogen attacks and/or insect injuries [2],[12],[13]. The antioxidant hydrophilic phenolic alcohols of VOO and their secondary metabolites also contribute to the long oil shelf-life and influence several organoleptic characteristics, including taste (e.g. bitter, astringent, pungent, throat-catching) and color [14]-[16].

**Table 1:** The main phenolic compounds in virgin olive oil

**Hydrophilic**		**Lipophilic**
**Phenolic alchols**	**Flavonoids**	**Tocopherols**
Hydroxytyrosol	Apigenin	(α, β, γ, δ)
Tyrosol	Luteolin	
**Secoroidoids**	**Phenolic acids**	**Tocotrienols**
Oleuropein	Gallic acid	(α, β, γ, δ)
Ligstroside aglycon	Vanillic acid	
**Lignans**	Benzoic acid	
(+)-1-pinoresinol	Cinnamic acid	
(+)-1-acetoxypinoresinol	Caffeic acid	
	Coumaric acid	

**Figure 2:**
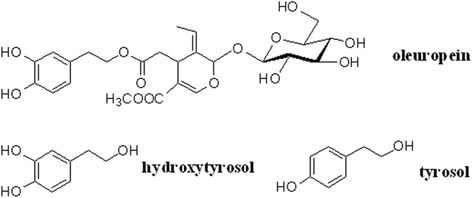
Chemical structure of the best known phenolic compounds in the VOO.

Nutraceutical properties have been attributed to secoiridoid oleuropein (OL) and its derivatives, the main alcohols 3,4-dihydroxyphenyl ethanol, also known as hydroxytyrosol (HT) and *p*-hydroxyphenyl ethanol or tyrosol [2],[11] (Figure 2). Such compounds are released from the olive fruit to VOO during the extraction process. In particular, OL is abundant in high amounts in unprocessed olive leaves and fruit, while higher concentration of HT may be found in the fruit and in olive oil, owing to chemical and enzymatic reactions that in the plant occur during maturation of the fruit [14],[17]. In addition, many agronomic factors, as cultivar, ripening stage, geographic origin of olive fruit and olive trees irrigation, as well as various oil extraction conditions during crushing, malaxation and VOO separation, may influence their final concentration in VOO [18]-[20].

**Figure 3:**
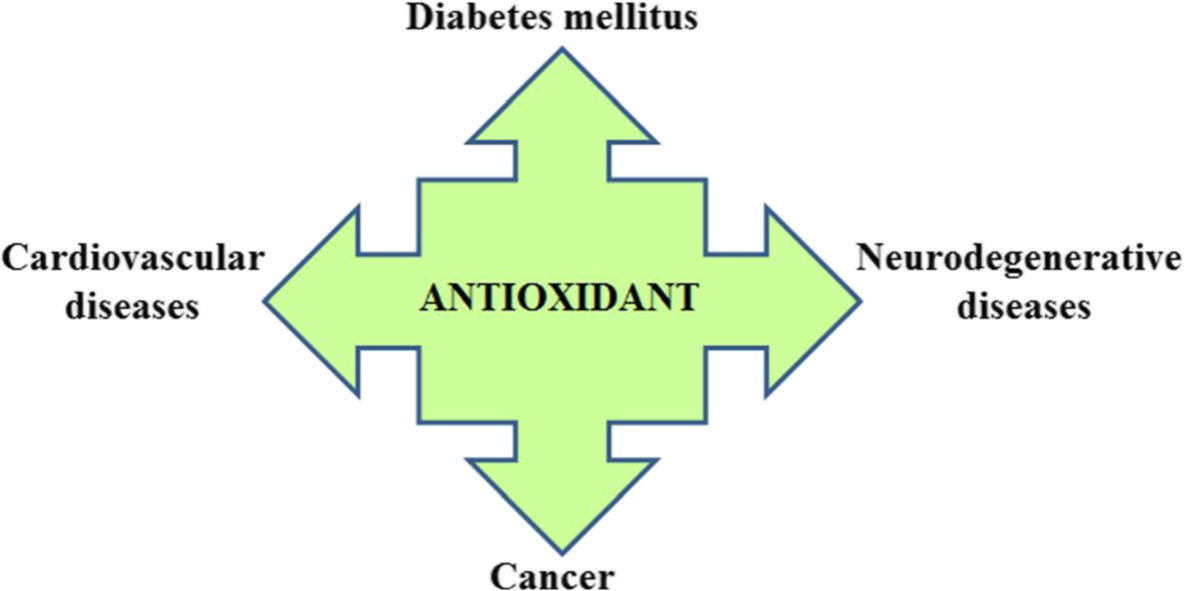
Antioxidant activity and related effects of oleuropein and hydroxytyrosol.

OL and HT represent the molecules of major interest for their biological and pharmacological properties, and, with no doubt, are among the most investigated antioxidant natural compounds [5],[11],[21]. They have been studied as isolated compounds or as components of ‘oil phenolic extracts’, showing a wide variety of beneficial effects, mainly related to their antioxidant activity (Figure 3), in many preclinical models of diseases [5],[22]-[26].

In the following sections will be described the biological effects of OL and HT, as resulting by *in vitro* and *in vivo* experimental data obtained with isolated compounds.

### Antioxidant activity of Oleuropein and Hydroxytyrosol

Defense against reactive oxygen species (ROS) is fundamental to protect cellular molecules as lipids, proteins or DNA and avoid the development of degenerative diseases. When the defensive mechanisms are overtaken by the action of the free radicals, the subsequent cellular damage may lead to several diseases, including atherosclerosis, cardiovascular diseases, skin and neurodegenerative diseases, diabetes mellitus and metabolic syndrome. Finally, physiological processes such as aging have been associated with a disequilibrium between the action of ROS and that of antioxidants [27],[28].

Antioxidant agents are present in various amount in several types of food. In the VOO, phenolic compounds in general, and OL derivatives in particular, act as natural antioxidants. They are important for the food stability and protect against the oxidation occurring naturally during VOO storage owing to reaction with air [29].

The antioxidant activity of OL and HT *in vivo* is related to their highly bioavailability [23],[24]: various studies have documented a high degree of absorption, fundamental to exert their metabolic and pharmacokinetics properties [23],[24],[30].

OL and HT behave as antioxidant acting as: a. free radical scavengers and radical chain breaking; b. anti-oxygen radicals; c. metal chelators. With their catecholic structure, they are able to scavenge the peroxyl radicals and break peroxidative chain reactions producing very stable resonance structures [2],[31].

A decrease in ROS production, derived by iron or copper induced oxidation of low-density lipoproteins (LDL), was first described after treatment with either OL or HT in an *in vitro* model, suggesting a chelating action on such metals [25],[32].

However, a strong free-radical scavenging action has been demonstrated also by using metal-independent oxidative systems [33] or measuring stable free radicals, such as 2,2-diphe-1-picrylhydrazyl (DPPH) [34],[35]. The ability to scavenge or reduce the generation of ROS was further confirmed both in leukocytes treated with phorbol 12-myristate 13-acetate (PMA) and in hypoxanthine/xanthine oxidase cell-free system through a chemiluminescence method [36],[37]. Again, a scavenging effect of OL and HT was demonstrated with respect to hypochlorous acid (HOCl) [36], a potent oxidant produced *in vivo* at the site of inflammation: this activity was demonstrated in a model of HOCl-mediated inactivation of catalase. This last evidence may have important implication in the protection from atherosclerosis, since HOCl can oxidize the apoproteic component of LDL (see next section). Zhu et al. have reported that HT induces simultaneously both phase II detoxifying enzymes (a set of important enzymes for protecting against oxidative damage) and mitochondrial biogenesis, two critical pathways occurring in the fight against oxidative stress [38]. An additional important element that contributes to the accumulation of intracellular ROS is the endoplasmic reticulum (ER) stress [39]: recently, it has been reported that HT is able both to modulate an adaptive signaling pathway activated after ER stress and to ameliorate ER homeostasis [40]. It must be noted that, at higher doses, OL and HT may exert pro-oxidant activity [41]-[44], responsible for the antiproliferative properties on cancer cells (see Section “Other activities”).

### Protection against cardiovascular diseases

Several studies have emphasized the importance of a regular use of olive oil in the benefits of traditional mediterranean diet on cardiovascular diseases [6],[45]-[47]. In particular, beside the antioxidant activity, vasodilatatory, anti-platelet aggregation and anti-inflammatory effects have been assigned to olive oil phenolic compounds such as OL and HT [5],[22],[23],[48].

Several reports have described the protective effects against atherosclerosis of OL and HT in preclinical experimental models. Visioli et al. [25] have demonstrated that OL and HT inhibit copper sulphate-induced oxidation of LDL. As previously mentioned, OL and HT exert a scavenging effect towards HOCl, which acts through chlorination of apoB-100 as an initiating agent in LDL lipid peroxidation [49], and this effect determines a retard in the onset of the atherosclerotic damage. In addition, Jemai et al. demonstrated that in rats fed with a cholesterol-rich diet, the same compounds were able to promote hypocholesterolemia, lowering LDL plasma levels and total cholesterol; also, they increased the levels of high-density lipoproteins (HDL) and the activity of antioxidant enzymes reducing LDL oxidation [50],[51]. Recently, the European Food Safety Authority (EFSA) has recognized protective effects of the olive oil phenolic compounds on LDL oxidation, in particular of HT [52].

Effects other than the reduction of LDL and cholesterol may explain the anti-atherogenic action of OL and HT, too (see Table 2). Carluccio et al. described the inhibition of endothelial activation, an early step in atherogenesis, by OL and HT, able to reduce lipopolysaccharide (LPS)-stimulated expression of vascular adhesion molecule-1 (VCAM-1) in human vascular endothelial cells by inhibition of its mRNA levels, thus decreasing monocyte cell adhesion to endothelial cells [53]. Two additional mechanisms involved in the vascular damages, platelet aggregation and proliferation of smooth muscle cells, are also antagonized by the olive oil phenolic compounds. It has been observed that HT inhibits *in vitro* platelet aggregation induced by thromboxane B2 production and collagen [54]. The same effect was observed in healthy rats assigned to diet supplemented with HT [55]: in this study was proposed that both an inhibition of cyclooxygenase (COX)-2 with a related decrease of thromboxane A2 blood levels and an increase of vascular nitric oxide production may contribute to this effect [55]. Inhibition of vascular smooth muscle cell proliferation has been demonstrated after treatment with OL, associated with a reduction of the extracellular regulated kinase-1/2 activity [56].

**Table 2:** Effects and mechanisms involved in the cardiovascular protection of oleuropein and hydroxytyrosol

**Protection**	**Effect**	**Mechanism**	**References**
Vascular disease	antioxidant	**↓**LDL oxidation	[24],[49],[50]
		**↓**Lipid peroxidation	[51]
	↓ endothelial activation	**↓**VCAM-1	[53]
	↓ monocyte adhesion		
	↓ platelet aggregation	n.d.	[54]
		↓COX-2 activity	[55]
		↓thromboxane A2	
		↑NO	
	↓ VSME proliferation	↓ERK 1/2 phosphorylation	[56]
Heart disease	↓ coronary occlusion (*)	**↓**CK activity	[57]
		**↓**GSSG	
	↓ cardiotoxicity (**)	↑AMPK phosphorylation	[58],[59]
		↓iNOS expression	
		**↑**METC activity	
	ischemia	hypolipidemia	[61]
		↓SOD activity	
	myocardial infarction	**↑**Akt phosphorylation	[62]
		**↑**eNOS phosphorylation	
		**↑**FOXO3a phosphorylation	

(*) myocardial injury induced by ischemia; (**) DXR-induced toxicity; n.d.: not determined.

*Abbreviations*: *AMPK* 5’-AMP-activated protein kinase; *CK* Creatine kinase; *COX* cyclooxygenase; *DXR* doxorubicin; *eNOS* endothelial nitric oxide synthase; *ERK* extracellular regulated kinase; *GSSG* Oxidize glutathione; *iNOS* inducible nitric oxide synthase; *LDL* low-density lipoproteins; *MMP* matrix metalloproteinases; *METC* mitochondrial electron transport chain; *NO* nitric oxide; *PDE* phosphodiesterase; *SOD* superoxide dismutase; *VCAM* vascular adhesion molecule; *VSME* vascular smooth muscle cells.

Some data exist also abut direct cardioprotective effects of these molecules. Manna et al. [57] analyzed OL effects in myocardial injury induced by ischemia; in isolated rat heart perfused with OL before induction of ischemia, were measured the levels of creatine kinase, a biochemical marker of cellular damage, and those of oxidize glutathione, a marker of heart exposure to oxidative stress and a key factor in the pathogenesis of atherosclerosis. OL significantly decreased levels of both markers suggesting a cardioprotective effect in the acute events that follow coronary occlusion. Recently, it has been observed that OL is able to prevent cardiomyopathy in rats treated with doxorubicin (DXR) [58]. In addition, Granados et al. have reported that HT attenuated DXR-associated chronic cardiac toxicity in rats with breast cancer ameliorating mitochondrial dysfunction [59].

The impact of OL was studied also *in vivo* in normal and hypercholesterolemic rabbits subjected to ischemia and reperfusion [62]. Treatment with OL for 3 or 6 weeks considerably reduced the infarct size in normal rabbits and, at higher doses, in hypercholesterolemic rabbits. Moreover, OL protection of re-perfused myocardium was associated with decreased total cholesterol and triglyceride levels [62].

The cardioprotective effects of HT have been confirmed in a study conducted with cardiomyocytes extracted from rats treated with this phenol. In these animals, administration of HT reduced the expression of proteins related to ageing as well as the infarct size and cardiomyocyte apoptosis [60].

In another study, a reduced infarct size with improvement in the myocardial function was shown in tyrosol-treated rats compared to non-treated controls [61].

### Protection against diabetes and metabolic disorders

In the early 90s, Gonzalez et al., using an animal model of alloxan-induced diabetes mellitus, first postulated a protective role of OL extracted by olive leaves [63]. Subsequent studies evidenced a strong link of the antidiabetic action with the antioxidant effects of OL. By treating alloxan-diabetic rabbits with OL, Al-Azzawie and Alhamdani obtained a significant hypoglycemic effect as compared with diabetic control animals, associated with restoration of the levels of malondialdehyde and most of the enzymatic and non-enzymatic endogenous antioxidants [64]. Similar data were reported in alloxan-diabetic rats treated with OL and HT from olive leaves [65] or using purified HT from olive mill waste both *in vitro* and in rats [66].

A close relationship between antioxidant and hypoglycemic activity of olive leaf extracts (OLE) was confirmed by Poudyal et al. [67] in rats with a diet-induced model of the metabolic syndrome. Supplementation of the diet with OLE enriched with OL and HT attenuated the metabolic alterations, including plasma glucose, triglyceride and total cholesterol concentrations. Such effects were paralleled by reduced plasmatic malondialdehyde and uric acid levels, therefore suggesting again a role for the antioxidant activity.

In another animal model of high-fat-diet (HFD)-induced obesity, hyperglycemia, hyperlipidemia, and insulin resistance, Cao et al. demonstrated the protective effect of HT, showing its ability to decrease HFD-induced lipid deposits through inhibition of the SREBP-1c/FAS pathway in liver and skeletal muscle tissues, enhance antioxidant enzyme activities, normalize expression of mitochondrial complex subunits and mitochondrial fission marker Drp1, and eventually inhibit apoptosis activation [68]. In addition, in mutant diabetic (db/db) mice, HT significantly decreased fasting glucose, and lipid serum levels, the latter effects obtained when treatment with metformin failed. As in the HFD model, muscle mitochondrial carbonyl protein levels and improved mitochondrial complex activities were also observed in db/db mice treated with HT [68]. Thus, at least for HT, the metabolic effects may be not limited to the action against the oxidative stress [68]. Moreover, in diabetic rats treated with HT, a reduction of the content of triglycerides and LDL-cholesterol and an increase of HDL-cholesterol levels has been reported [26]. Recently, El et al. suggested that improvement of glucose-induced insulin release as well as increased peripheral uptake of glucose are both involved in the hypoglycemic effect of OL [22].

The effects of OL and HT on insulin action have recently been demonstrated by De Bock et al. in overweight middle-aged men: administration of a diet supplemented with olive leaf polyphenols (51.1 mg OL, 9.7 mg HT for day) determined both amelioration of insulin action and secretion, two aspects of glucose regulation. Such an effect was independent of fat distribution, dietary intakes and physical activity and was comparable to that seen with drugs used to treat diabetes [69].

Interesting results have come from elucidation of the gene expression profile performed in the liver of obese mice treated with OL [70]. In particular, the mRNA levels of lipocalin 2 (LCN2) (0.33-fold) resulted down-regulated after OL treatment [70]. Since LCN2 deficiency in mice has been associated with protection from developing aging-and obesity-associated insulin resistance and hyperglycemia [71], the effect on this protein may represent an additional target of OL action.

### Other activities

OL and HT displayed protective effects against several other diseases, mainly dependent on their antioxidant activity. Protection against the genotoxic action of the ROS is one of the mechanisms explaining the anticancer effects of these compounds [72]-[74]. In addition, OL and HT may act also through the modulation of pro- and anti-oncogenic signaling pathways, leading to cell apoptosis and growth arrest of several tumor cell lines *in vitro*[73]-[80]. It has been recently suggested that the antiproliferative and pro-apoptotic effects of OL and HT on tumor cells, may be mediated by their capability to induce the accumulation of hydrogen peroxide in the culture medium [41]-[44].

At present, there are few studies demonstrating the block of tumor growth *in vivo*[24],[79]. Results from a recent work by Sepporta et al. [81] demonstrated that OL was able to inhibit the MCF-7 human breast cells xenograft growth and their invasiveness into the lung.

Protective properties against infections are attributed to olive oil extracts or isolated compounds, as confirmed by many studies reporting anti-microorganisms and anti-virus activity [82]-[86].

By acting against oxidation, inflammation and atherosclerosis, HT, OL and derivatives result effective also in age-related disorders, as neurodegenerative diseases [48],[87],[88]. Neuroprotection may derive by interference with amyloid beta peptide (Aβ) and Tau protein aggregation [87]-[90]. Furthermore the potential neuroprotective effects of HT and OL have also been reported against brain damages such as brain hypoxia-reoxygenation, cerebral ischemia and spinal cord injury [91],[92].

At the skin level, HT conjugates with fatty acids showed optimal topical delivery features through the human stratum corneum and viable epidermis membranes [93]. Moreover, co-administration of HT and hydrocortisone in the co-loaded nanoparticles provide additional anti-inflammatory and antioxidant benefits in atopic dermatitis treatment [94]. OL intra-dermal injection also reduced cell infiltration in the wound site and forwards collagen fibers deposition and more advanced re-epithelialization *in vivo*[95].

Finally, OL has demonstrated beneficial antioxidant properties even against ethanol-induced gastric damages *in vivo*[96].

Table 3 summarizes the main results regarding the effects of isolated OL and HT in preclinical models of neoplastic, neurodegenerative and skin diseases.

**Table 3:** Effects of oleuropein and hydroxytyrosol in neoplastic, neurodegenerative and skin diseases

**Protection**	**Model**	**Effect**	**Mechanism**	**References**
Cancer	Bladder cancer cells	↓cell proliferation	n.d.	[75]
	Breast cancer cells	↓cell proliferation	↓Cyclin D1, ↓Pin1,	[42],[43],[73],[75],[78],[79],[97]
		cell cycle arrest	↑c-jun, ↑H_2_O_2_	
		↓motility and invasiveness		
		↑apoptosis		
		↓DNA damage		
	Colon cancer cells	↓cell proliferation	↑H_2_O_2_	[42],[79]
		↑apoptosis		
		↓motility and invasiveness		
	Glia cancer cells	↓motility	n.d.	[79]
	Leukemia cells	↓cell proliferation	↑H_2_O_2_, ↑Cyclin D3,	[41],[72],[76]
		cell cycle arrest	↓CDK6	
		↑apoptosis		
		↓DNA damage		
	Prostate cancer cells	↓cell proliferation	↑H_2_O_2_, ↑O_2_^**−**^	[42],[44]
		↑apoptosis		
	Renal cancer cells	↓cell proliferation	n.d.	[79]
	Thyroid cancer cells	cell cycle arrest	↓pAKT, ↓pERK	[80]
		↑apoptosis		
	Mice xenograft and albino hairless HOS:HR-mice	↓tumor growth	↓COX-2, ↓VEGF, ↓MMP-2/9/13	[81],[98]
Neurodegenerative disease	Brain hypoxia-reoxygenation	antioxidant ↓inflammation	↓lipid peroxidation, ↓PGE2, ↓IL1β, ↓NO	[92]
	*In vitro* assay	↓Tau aggregation	n.d.	[90]
	Sprague–Dawley rats	↓spinal cord injury	↓MDA activity	[91]
Skin disease	Albino hairless HOS:HR and C57BL/6J mice	↑UVB protection	↓MMP-13	[98],[99]
	Balb/c mice	↑wound healing	↑VEGF	[95]

*Abbreviations*: *AKT* serine/threonine-specific protein kinase; *CDK* cyclin-dependent protein kinase; *c-jun* transcription factor member; *COX-2* cyclooxygenase-2; *H*_*2*_*O*_*2*_ hydrogen peroxide; *ERK* extracellular regulated kinase; *IL1β* interleukin 1β; *MDA* malondialdehyde; *MMP-2/9/13* matrix metalloproteinase; *NO* nitric oxide; *O*_*2*_ superoxide ions; *Pin1* peptidyl-prolyl cis–trans isomerase; *PGE2* prostaglandin E_2_; *VEGF* vascular endothelial growth factor; *n.d.* not determined.

## Conclusions and remarks

The large number of preclinical studies described herein has revealed the molecular basis of the beneficial actions of single components of the phenolic fraction of olive oil. Although some of these effects may derive from the interaction of the various VOO components generated by enzymatic hydrolysis of the phenolic extracts when used as a mixture, OL and HT are considered the major candidates for a pharmacological use, both as single drug or after enrichment of olive oil or other food components. Moreover, OL and HT possess high bioavailability [23],[24], together with an absolute absence of either acute or sub-chronic toxicity, at least as shown in animal experimental models [100],[101]. In view of a possible use of OL and HT in human pathology, more than one approach is under investigation. The high stability and bioavailability of these compounds has encouraged attempts to enrich the olive oil or other food components with isolated/purified phenolic compounds [102],[103]. In addition, implementation of the preparation process by the food industry and modification of the molecules to obtain more active derivatives are also promising strategies. Noteworthy, recent results obtained with OL aglycone or some semisynthetic derivatives [80],[97],[102],[104]-[107] suggest that it is possible to improve the pharmacological properties of these compounds. Further studies will better clarify the *in vivo* effects of OL, HT and their semisynthetic derivatives, to use as individual agents or in combination, with particular attention to their safety profile on humans, and open the way to a wide utilization in human pharmacology.

## Abbreviations

COX: Cyclooxygenase

DXR: Doxorubicin

DPPH: 2,2-diphe-1-picrylhydrazyl

EFSA: European food safety authority

ER: Endoplasmic reticulum

HDL: Low-density lipoproteins

HFD: High-fat-diet

HT: Hydroxytyrosol

HOCl: Hypochlorous acid

LDL: Low-density lipoproteins

LCN2: Lipocalin 2

LPS: Lipopolysaccharide

MMP: Matrix metalloproteinases

MUFA: Monounsaturated fatty acids

PMA: Phorbol 12-myristate 13-acetate

OL: Oleuropein

OLE: Olive leaf extracts

ROS: Reactive oxygen species

VCAM-1: Vascular adhesion molecule-1

VOO: Virgin olive oil

## Competing interests

The authors declare that there are no competing interests.

## Authors’ contributions

DR and AP contributed to the conception of the idea, drafted the manuscript and critically reviewed the final manuscript; DR elaborated the sections Introduction and Conclusion and editing the manuscript; SB elaborated the section Antioxidant activity of Oleuropein and Hydroxytyrosol, the figures, the tables and editing the manuscript; TM and AP elaborated the section Protection against Cardiovascular Diseases; MC elaborated the section Protection against Diabetes and Metabolic disorders and the figures; SL elaborated the section Other Activities. All authors read and approved the final manuscript.
